# Polymicrobial bloodstream infections per se do not increase mortality compared to monomicrobial bloodstream infections in sepsis patients: a Korean nationwide sepsis cohort study

**DOI:** 10.1186/s12879-024-09130-5

**Published:** 2024-03-05

**Authors:** Su Yeon Lee, Mi Hyeon Park, Dong Kyu Oh, Chae-Man Lim, on behalf of the Korean Sepsis Alliance (KSA) investigators, Sang-Bum Hong, Gee Young Suh, Kyeongman Jeon, Ryoung-Eun Ko, Young-Jae Cho, Yeon Joo Lee, Sung Yoon Lim, Sunghoon Park, Jeongwon Heo, Jae-myeong Lee, Kyung Chan Kim, Youjin Chang, Sang-Min Lee, Suk-Kyung Hong, Woo Hyun Cho, Sang Hyun Kwak, Heung Bum Lee, Jong-Joon Ahn, Gil Myeong Seong, Song-I. Lee, Tai Sun Park, Su Hwan Lee, Eun Young Choi, Jae Young Moon, Hyung Koo Kang

**Affiliations:** Department of Pulmonary and Critical Care Medicine, Asan Medical Center, University of Ulsan College of Medicine, 88 Olympic-Ro 43-Gil, Songpa-Gu, Seoul, 05505 South Korea

**Keywords:** Bacteremia, Fungemia, Sepsis, Mortality, Coinfection

## Abstract

**Background:**

There is limited information about the outcomes of polymicrobial bloodstream infections in patients with sepsis. We aimed to investigate outcomes of polymicrobial bloodstream infections compared to monomicrobial bloodstream infections.

**Methods:**

This study used data from the Korean Sepsis Alliance Registry, a nationwide database of prospective observational sepsis cohort. Adult sepsis patients with bloodstream infections from September 2019 to December 2021 at 20 tertiary or university-affiliated hospitals in South Korea were analyzed.

**Results:**

Among the 3,823 patients with bloodstream infections, 429 of them (11.2%) had polymicrobial bloodstream infections. The crude hospital mortality of patients with sepsis with polymicrobial bloodstream infection and monomicrobial bloodstream infection was 35.7% and 30.1%, respectively (*p* = 0.021). However, polymicrobial bloodstream infections were not associated with hospital mortality in the proportional hazard analysis (HR 1.15 [0.97–1.36], *p* = 0.11). The inappropriate use of antibiotics was associated with increased mortality (HR 1.37 [1.19–1.57], *p* < 0.001), and source control was associated with decreased mortality (HR 0.51 [0.42–0.62], *p* < 0.001).

**Conclusions:**

Polymicrobial bloodstream infections per se were not associated with hospital mortality in patients with sepsis as compared to monomicrobial bloodstream infections. The appropriate use of antibiotics and source control were associated with decreased mortality in bloodstream infections regardless of the number of microbial pathogens.

**Supplementary Information:**

The online version contains supplementary material available at 10.1186/s12879-024-09130-5.

## Background

Bloodstream infection is a major cause of morbidity and mortality and carries a high burden in terms of healthcare costs [1, 2]. Among the patients who were diagnosed with sepsis or septic shock, bloodstream infections account for approximately 40% of the cases [3, 4]. Polymicrobial bloodstream infections, which refer to infection caused by more than two pathogens detected in blood culture tests, account for 10% of the total bloodstream infections [5]. But there was limited information about the outcomes of polymicrobial bloodstream infections compared to those of monomicrobial bloodstream infection in patients with sepsis or septic shock. Previous studies on polymicrobial bloodstream infections have shown conflicting data about mortality [6–10]. However, these studies had limitations; they were confined to patients with either hematological or solid malignancies [6, 7], limited to patients admitted to intensive care units [8], or were underpowered due to small sample sizes [9, 10]. Therefore, in this study, we aimed to compare the outcomes of polymicrobial bloodstream infections with monomicrobial bloodstream infections by the nationwide sepsis cohort in Korea. Furthermore, we sought to identify risk factors associated with polymicrobial bloodstream infections.

## Materials and methods

### Study design and patients

This study used data from the KSA Registry, a nationwide database of prospective observational cohort. In this registry, we registered patients with sepsis from September 2019 to December 2021 at 20 tertiary or university-affiliated hospitals in South Korea. The patients aged 19 years or older and diagnosed with sepsis or septic shock according to the Sepsis-3 definition in a general ward or an emergency department of participating hospitals during the study period were enrolled [11]. We defined community-onset sepsis as the patients were in emergency department (ED), and hospital-onset sepsis as the patients were in general ward and fulfilled inclusion criteria (Appendix 1) and sepsis-3 criteria, respectively. After patient registration, the patient was followed up until the patient died or was discharged from hospital. In this sepsis registry, a bloodstream infection associated with a sepsis event was predefined when bacteria or fungus were cultured in blood culture tests conducted within 48 h before and after time zero. A polymicrobial bloodstream infection was defined when two or more bacteria or fungi were cultured simultaneously, whereas a monomicrobial bloodstream infection was defined when a single bacteria or fungus was cultured in blood culture tests. The principal investigator at each participating institution was responsible for determining whether the cultured organisms were contaminants, such as resident flora, or true sepsis-causing pathogens. The determination was based on several factors, including the type of organism cultured, the number of positive blood culture bottles, the time to detectable growth, the amount of growth in each culture bottle, clinical and laboratory evidence, and the origin of the cultures, such as whether they were obtained from a catheter or a peripheral site [12]. Only organisms identified as true pathogens were collected and recorded. Of the 11,981 patients from the total sepsis cohort, 28 patients did not undergo blood culture tests. Among the 11,953 patients, 3,823 patients were diagnosed with bloodstream infections. We compared patients with polymicrobial bloodstream infections (*n* = 429) to those with monomicrobial bloodstream infections (*n* = 3,394) regarding clinical characteristics, predisposing factors, and mortality.

### Data collection

The electronic medical records of all the eligible patients were reviewed by study personnel in each participating hospital, and data were collected using a standardized case report form. The following information were collected: demographic data, underlying comorbidities, disease severity scores, hospital outcomes, received treatment, and serial physiologic data (Appendix 2). The following areas were investigated as potential sources of infection: pulmonary, abdominal, urinary, skin/soft tissue, catheter-related, neurologic, or systemic infections without clear primary site. The appropriateness of antibiotic use in our study was evaluated under two scenarios. First, when culture and antibiotic susceptibility test results were available, we assessed antibiotic use appropriateness based on these findings. Second, in instances where the causative organism was unidentified and susceptibility results were unavailable, appropriateness was determined based on the empirical antibiotic use as recommended by standard clinical guidelines. For instance, for patients with community-acquired pneumonia where no organism was detected, the administration of a third-generation cephalosporin or respiratory quinolone was deemed appropriate. A multidrug-resistant (MDR) pathogen was considered to be present if the following pathogens were resistant to more than three classes of drugs: *Staphylococcus aureus*, *Enterococcus* spp., Enterobacteriaceae, *Pseudomonas aeruginosa*, and *Acinetobacter* spp. [13]. Information on methicillin-resistant *Staphylococcus aureus* (MRSA), vancomycin-resistant *Enterococcus*, extended-spectrum-beta-lactamase–producing Enterobacteriaceae, carbapenem-resistant *Enterobacteriaceae*, carbapenem-resistant *Pseudomonas aeruginosa*, and carbapenem-resistant *Acinetobacter baumannii* was also collected.

### Study outcomes

The primary outcome was hospital mortality of polymicrobial bloodstream infection. The secondary outcomes were 28-day mortality, ICU admission, ICU mortality between monomicrobial and polymicrobial bloodstream group, and predisposing factors of polymicrobial bloodstream infection.

### Statistical analysis

Variables are presented either as means with standard deviations or medians with an interquartile range, as appropriate. The Student’s *t*-test was used to compare the continuous variables, and the chi-squared or Fisher’s exact tests were used to compare the categorical variables. The risk factors that correlated with the occurrence of polymicrobial bloodstream infection were identified using multivariate logistic regression analysis. A Cox proportional hazards model was used to identify the risk factors that correlated with hospital mortality. Variables with *p*-values of < 0.10 on univariate analysis were selected, and backward elimination method was used in multivariate analysis. All the *p*-values were two-tailed, and statistical significance was set at a *p*-value of < 0.05. R programming (open software) was used for all the statistical analyses and graphs.

## Results

Of the 11,953 patients with sepsis or septic shock who underwent blood cultures in the registry, 3,823 (32.0%) patients were diagnosed with bloodstream infections. Among the 3,823 patients with bacteremia or fungemia, 429 (11.2%) patients had polymicrobial bloodstream infections and 3,394 (88.8%) patients had monomicrobial bloodstream infection. In sepsis patients with polymicrobial bloodstream infections, BMI was lower (21.8 ± 3.9 vs. 22.2 ± 4.1, *p* = 0.033), and the proportion of hospital-onset sepsis tended to be higher (23.8% vs. 20.2%, *p* = 0.098) than those with monomicrobial bloodstream infection (Table 1). The patients admitted in the surgical ICU were more diagnosed with polymicrobial bloodstream infections than those admitted in the medical ICU (34.3% vs. 21.4%, *p* = 0.006). Those patients with polymicrobial bloodstream infections had more solid malignant tumors (53.6% vs. 37.5%, *p* < 0.001), and their Charlson comorbidity index was higher (6.2 ± 2.4 vs. 5.7 ± 2.4, *p* < 0.001) than those with monomicrobial bloodstream infection. Abdominal infections were more frequent (54.8% vs. 34.4%, *p* < 0.001) in patients with polymicrobial bloodstream infections, whereas pulmonary (26.5% vs. 19.6%, *p* = 0.002) and urinary infections (29.2% vs. 15.6%, *p* < 0.001) were less frequent compared to those with monomicrobial bloodstream infection. Those patients with polymicrobial bloodstream infections had a higher SOFA score (7.5 ± 3.2 vs. 6.9 ± 3.2, *p* < 0.001) and a higher lactate level (5.2 ± 3.5 vs. 4.4 ± 3.5 mmol/L, *p* < 0.001) at time zero. The proportion of septic shock was tended to be higher in patients with polymicrobial bloodstream infections (26.3% vs. 22.5%, *p* = 0.089).

**Table 1:** Characteristics of polymicrobial bloodstream infections vs. monomicrobial bloodstream infections in patients with sepsis

Variable	Monomicrobial bloodstream infections (*n* = 3394)	Polymicrobial bloodstream infections (*n* = 429)	*P*-value
Age, median(IQR)	73.0 (62.0–81.0)	72.0 (64.0–79.0]	0.297
Sex, n(%)	1850 (54.5)	253 (59.0)	0.089
BMI, mean ± SD	22.2 ± 4.1	21.8 ± 3.9	0.033
Community-onset sepsis, n(%)	2708 (79.8)	327 (76.2)	0.098
Hospital-onset sepsis, n(%)	686 (20.2)	102 (23.8)	0.098
Medical ICU	539 (78.6)	67 (65.7)	0.006
Surgical ICU	147 (21.4)	35 (34.3)	0.006
Comorbidity, n(%)			
Cardiovascular disease	763 (22.5)	91 (21.2)	0.594
Chronic lung disease	346 (10.2)	38 (8.9)	0.434
Chronic neurological disease	710 (20.9)	106 (24.7)	0.081
Chronic liver disease	397 (11.7)	48 (11.2)	0.819
Diabetes	1199 (35.3)	162 (37.8)	0.348
Chronic kidney disease	419 (12.3)	54 (12.6)	0.948
Connective tissue disease	83 (2.4)	14 (3.3)	0.394
Immunocompromised	114 (3.4)	22 (5.1)	0.084
Hematological malignancies	274 (8.1)	30 (7.0)	0.494
Solid malignant tumors	1274 (37.5)	230 (53.6)	< 0.001
Charlson comorbidity index, mean ± SD	5.7 ± 2.6	6.2 ± 2.4	< 0.001
ECOG > = 2, n(%)	2030 (59.8)	274 (63.9)	0.117
CFS score, mean ± SD	5.1 ± 2.2	5.2 ± 2.2	0.117
Source of infection, n(%)			
Pulmonary	899 (26.5)	84 (19.6)	0.002
Abdominal	1166 (34.4)	235 (54.8)	< 0.001
Urinary	992 (29.2)	67 (15.6)	< 0.001
Skin and soft tissue	145 (4.3)	19 (4.4)	0.981
Catheter-related	86 (2.5)	10 (2.3)	0.929
Systemic infections	369 (10.9)	38 (8.9)	0.233
Neurologic	15 (0.4)	4 (0.9)	0.319
Initial septic shock, n(%)	765 (22.5)	113 (26.3)	0.089
SOFA score at time zero, mean ± SD	6.9 ± 3.2	7.5 ± 3.2	< 0.001
Lactate, mmol/L, mean ± SD	4.4 ± 3.5	5.2 ± 3.5	< 0.001

*IQR* Interquartile range, *SD* Standard deviation, *ICU* Intensive care unit, *ECOG* European Cooperative Oncology Group, *CFS* Clinical Frailty scale, *SOFA* Sequential Organ Failure Assessment

*Escherichia coli* was the most common causative pathogen found in patients with polymicrobial bloodstream infections (50.3%), followed by *Klebsiella pneumoniae* (33.3%), *Enterococcus faecium* (14.5%), and *Enterococcus faecalis* (11%) (Table 2). The most common combination was *Escherichia coli* and *Klebsiella pneumoniae* (13.8%), followed by *Escherichia coli* and *Enterococcus faecium* (3.5%), and *Escherichia coli* and *Enterococcus faecalis* (3.0%) (eFigure 1). In those patients with monomicrobial bloodstream infection, the most common pathogen found was *Escherichia coli* (37%), followed by *Klebsiella pneumoniae* (19.1%), *Staphylococcus aureus* (8.1%), and *Pseudomonas aeruginosa* (4%). MDR pathogens were more common in patients with polymicrobial bloodstream infections (46.6% vs. 33.8%, *p* < 0.001), and fungemia was detected twice common in patients with polymicrobial bloodstream infections (4.4% vs. 2.0%, *p* = 0.002). Supplementary eTables 1 and 2 detail the gram-positive and gram-negative bacteria classified as “Others” in Table 2.

**Table 2:** Microbiology of bloodstream infection

Variable	Monomicrobial bloodstream infections (*n* = 3394)	Polymicrobial bloodstream infections (*n* = 429)	*P*-value
Gram-positive bacteremia, n(%)	818 (24.1)	236 (55.0)	< 0.001
*Staphylococcus aureus*	276 (8.1)	34 (7.9)	0.957
*Streptococcus pyogenes*	6 (0.2)	0 (0.0)	0.823
*Streptococcus agalactiae*	23 (0.7)	1 (0.2)	0.439
*Streptococcus pneumoniae*	30 (0.9)	1 (0.2)	0.258
*Enterococcus faecalis*	54 (1.6)	47 (11.0)	< 0.001
*Enterococcus faecium*	78 (2.3)	62 (14.5)	< 0.001
*Listeria monocytogenes*	3 (0.1)	0 (0.0)	1
*Corynebacterium striatum*	21 (0.6)	6 (1.4)	0.131
Non–*S. aureus staphylococcus* spp.	141 (4.2)	43 (10.0)	< 0.001
Others	186 (5.5)	82 (19.1)	< 0.001
Gram-negative bacteremia, n(%)	2488 (73.3)	385 (89.7)	< 0.001
*Escherichia coli*	1256 (37.0)	216 (50.3)	< 0.001
*Klebsiella pneumoniae*	649 (19.1)	143 (33.3)	< 0.001
*Klebsiella oxytoca*	36 (1.1)	32 (7.5)	< 0.001
*Klebsiella* (*Enterobacter*) *aerogenes*	26 (0.8)	14 (3.3)	< 0.001
*Pseudomonas aeruginosa*	136 (4.0)	44 (10.3)	< 0.001
*Acinetobacter baumannii*	47 (1.4)	15 (3.5)	0.002
*Citrobacter* spp.	40 (1.2)	28 (6.5)	< 0.001
*Enterobacter cloacae*	58 (1.7)	35 (8.2)	< 0.001
*Proteus* spp.	74 (2.2)	24 (5.6)	< 0.001
*Serratia marcescens*	21 (0.6)	5 (1.2)	0.324
*Neisseria meningitidis*	0 (0.0)	1 (0.2)	0.219
*Haemophilus influenzae*	4 (0.1)	0 (0.0)	1
*Stenotrophomonas maltophilia*	5 (0.1)	2 (0.5)	0.392
Others	137 (4.0)	60 (14.0)	< 0.001
Fungemia, n(%)	67 (2.0)	19 (4.4)	0.002
*Candida albicans*	31 (0.9)	5 (1.2)	0.807
*Candida glabrata*	11 (0.3)	9 (2.1)	< 0.001
*Candida parapsilosis*	5 (0.1)	1 (0.2)	1
*Candida tropicalis*	13 (0.4)	3 (0.7)	0.576
*Cryptococcus neoformans*	1 (0.0)	2 (0.5)	0.033
Others	4 (0.1)	1 (0.2)	1
MDR pathogen	1146 (33.8)	200 (46.6)	< 0.001

*Spp.* Species, *MDR* Multidrug resistant

In 183 patients (42.7%) with polymicrobial bloodstream infections, multiple gram-negative bacteria were cultured, and in 193 patients (45%), gram-negative and gram-positive bacteria were cultured (Table 3). Two microorganisms were grown in 83% of patients with polymicrobial bloodstream infections. Three or more microorganisms were grown in 17% of patients. Appropriate antibiotics were administered in 75.2% of patients who were diagnosed with gram-negative and gram-positive bacteremia and in 83.6% and 88.2% of patients with multiple gram-negative and multiple gram-positive bacteremia, respectively.

**Table 3:** Pathogens in patients with polymicrobial bloodstream infections

Variable	Polymicrobial bloodstream infections (*n* = 429)	Appropriate use of antibiotics
All gram-negative bacteria, n(%)	183 (42.7)	153 (83.6)
All gram-positive bacteria, n(%)	34 (7.9)	30 (88.2)
Gram-negative, gram-positive bacteria mixed, n(%)	193 (45.0)	145 (75.1%)
2 types of fungemia, n(%)	2 (0.5)	2 (100%)
Gram-negative bacteria, fungemia mixed, n(%)	8 (1.9)	5 (62.5)
Gram-positive bacteria, fungemia mixed, n(%)	8 (1.9)	7 (87.5)
Gram-negative, gram-positive bacteria and fungemia mixed	1 (0.2)	1 (100%)
Two pathogens identified, n(%)	356 (83.0)	288 (80.9)
Three pathogens identified, n(%)	60 (14.0)	48 (80)
Four pathogens identified, n(%)	13 (3.0)	7 (53.8)
MDR pathogen identified, n(%)	200 (46.6)	133 (66.5)
Mixed type of infection	210 (49.0)	158 (75.2%)

*MDR* Multidrug resistant

Nonsurgical source control including percutaneous drainage (PCD) was used more frequently in patients with polymicrobial bloodstream infection (22.1% vs. 14.4%, *p* < 0.001) (Table 4). The proportion of ICU admission and ICU length of stay was similar between the two groups. The proportion of patients who received mechanical ventilation (MV) (28.2% vs. 20.1%, *p* < 0.001) and the proportion of patients who received continuous renal replacement therapy (CRRT) (17.9% vs. 13.7%, *p* = 0.022) were higher in patients with polymicrobial bloodstream infection. The crude hospital mortality (35.7% vs. 30.1%, *p* = 0.021) and 28-day mortality (34.0% vs. 29.1%, *p* = 0.049) were higher in patients with polymicrobial bloodstream infection. Using the Cox proportional hazards model, we analyzed the factors associated with mortality in the entire patients with bloodstream infections (Table 5). Polymicrobial bloodstream infection per se was not associated with mortality (HR 1.15 [0.97–1.36], *p* = 0.11). The inappropriate use of antibiotics was associated with increased mortality (HR 1.37 [1.19–1.57], *p* < 0.001), and the undergoing source control was associated with decreased mortality (HR 0.51 [0.42–0.62], *p* < 0.001) in the entire patients with bloodstream infections. In patients with polymicrobial bloodstream infections, source control was associated with decreased mortality (HR 0.32 [0.19–0.54], *p* < 0.001), and preexisting severe fragility (HR 1.74 [1.03–2.90], *p* = 0.04) and primary bloodstream infection (HR 2.01 [1.26–3.20], *p* = 0.003) were associated with increased mortality (eTable 3).

**Table 4:** Treatment and outcomes of polymicrobial bloodstream infections vs. monomicrobial bloodstream infections in patients with sepsis

Variable	Monomicrobial bloodstream infections (*n* = 3394)	Polymicrobial bloodstream infections (*n* = 429)	*P*-value
Appropriate use of antibiotics, n (%)	2834 (83.7)	343 (80.3)	0.091
Inappropriate use of antibiotics, n (%)	552 (16.3)	84 (19.7)	0.091
Infection source control, n (%)	603 (17.8)	107 (24.9)	< 0.001
Surgical	119 (3.5)	14 (3.3)	0.905
Non-surgical	488 (14.4)	95 (22.1)	< 0.001
Percutaneous drainage	414 (12.2)	84 (19.6)	< 0.001
Pleural	22 (5.3)	0 (0.0)	0.062
Hepatobiliary	296 (71.5)	74 (88.1)	0.002
Peritoneal	31 (7.5)	5 (6.0)	0.791
Others	65 (15.7)	5 (6.0)	0.030
ICU admission, n (%)	1543 (45.5)	209 (48.7%)	0.221
ICU length of stay, days, median (IQR)	5.0 (3.0–8.0)	5.0 (3.0–10.0)	0.209
ICU mortality, n (%)	401 (26.0)	62 (29.7)	0.295
SAPS3, mean ± SD	74.5 ± 16.3	80.3 ± 15.6	< 0.001
MV apply, n (%)	681 (20.1)	121 (28.2)	< 0.001
MV duration, days, median (IQR)	4.0 (2.0–11.0)	5.0 (3.0–11.0)	0.105
CRRT apply, n (%)	466 (13.7)	77 (17.9)	0.022
ECMO apply, n (%)	12 (0.4)	3 (0.7)	0.503
Hospital length of stay, days, median (IQR)	15.0 (8.0–27.0)	17.0 (9.0–30.0)	0.025
Hospital mortality, n (%)	1021 (30.1)	153 (35.7)	0.021
28-day mortality, n (%)	903 (29.1)	135 (34.0)	0.049

*IQR* Interquartile range, *SD* Standard deviation, *ICU* Intensive care unit, *MV* Mechanical ventilation, *CRRT* Continuous renal replacement therapy, *ECMO* Extracorporeal membrane oxygenation

**Table 5:** Factors associated with mortality in patients with bloodstream infections by Cox proportional hazards analysis

Variable	Univariate			Multivariate		
	HR	95%CI	*P*-value	HR	95%CI	*P*-value
Age	1.00	1.00, 1.01	0.033			
Sex, male	1.16	1.03, 1.30	0.012			
BMI	0.99	0.97, 1.00	0.072			
Community-onset sepsis	0.99	0.87, 1.13	> 0.9			
Polymicrobial bloodstream infections	1.15	0.97, 1.36	0.11			
Cardiovascular disease	1.01	0.88, 1.15	0.9			
Chronic lung disease	1.11	0.92, 1.33	0.3			
Chronic neurological disease	0.81	0.70, 0.94	0.007	0.70	0.59, 0.82	< 0.001
Chronic liver disease	1.08	0.92, 1.28	0.3			
Diabetes	0.91	0.81, 1.03	0.12			
Chronic kidney disease	1.27	1.09, 1.49	0.002			
Connective tissue disease	0.71	0.48, 1.07	0.10			
Immunocompromised	1.10	0.84, 1.44	0.5			
Hematological malignancies	1.46	1.22, 1.74	< 0.001	1.46	1.20, 1.77	< 0.001
Solid malignant tumors	1.37	1.23, 1.54	< 0.001	1.30	1.12, 1.52	< 0.001
Charlson comorbidity index	1.08	1.06, 1.11	< 0.001	1.04	1.01, 1.07	0.005
ECOG score	1.29	1.24, 1.35	< 0.001	1.08	0.99, 1.19	0.10
CFS score	1.18	1.15, 1.22	< 0.001	1.14	1.08, 1.21	< 0.001
Source of infection						
Pulmonary	1.75	1.55, 1.98	< 0.001	1.41	1.22, 1.62	< 0.001
Abdominal	0.85	0.75, 0.96	0.010			
Urinary	0.51	0.44, 0.60	< 0.001	0.55	0.46, 0.65	< 0.001
Skin and soft tissue	1.07	0.83, 1.38	0.6			
Catheter-related	0.90	0.64, 1.26	0.5			
Systemic infections	1.63	1.39, 1.91	< 0.001	1.38	1.15, 1.66	< 0.001
Neurologic	0.48	0.18, 1.27	0.14			
Initial septic shock	1.56	1.38, 1.76	< 0.001	1.55	1.37, 1.76	< 0.001
Inappropriate use of antibiotics	1.60	1.39, 1.83	< 0.001	1.37	1.19, 1.57	< 0.001
Source control	0.45	0.38, 0.54	< 0.001	0.51	0.42, 0.62	< 0.001

*HR* Hazard ratio, *CI* Confidence interval, *BMI* Body mass index, *ECOG* European Cooperative Oncology Group, *CFS* Clinical Frailty scale

**Fig. 1:**
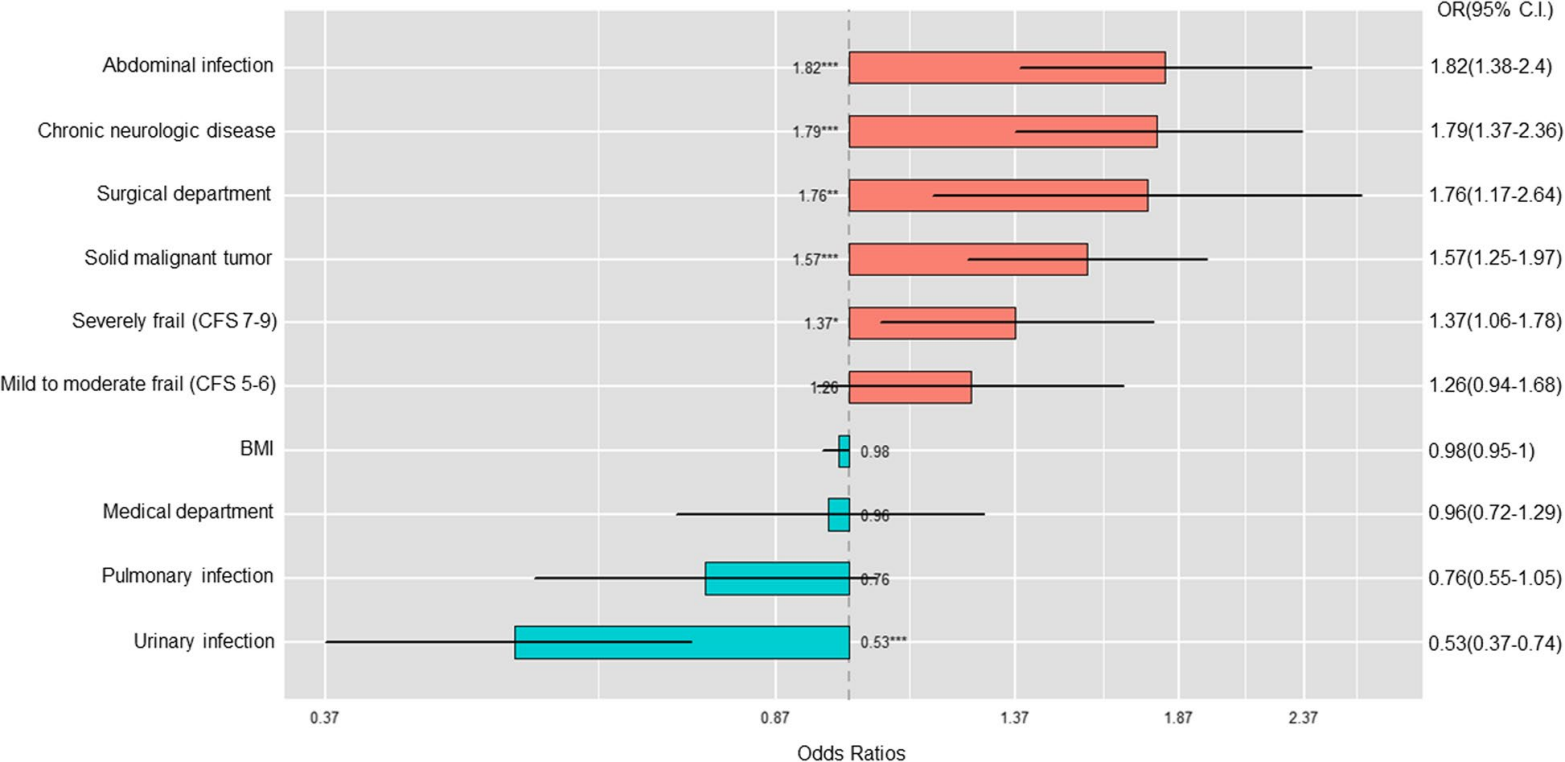
Factors associated with occurrence of polymicrobial bloodstream infections. (OR, odds; CI, confidence interval; BMI, body mass index; CFS, Clinical Frailty scale)

We used multivariate logistic regression analysis to analyze the factors associated with the development of polymicrobial bloodstream infections (Fig. 1, eTable 4). Surgical ICU admission was associated with increased risk of polymicrobial bloodstream infection (OR 1.76 [1.17–2.64], *p* = 0.0063). Chronic neurological disease (OR 1.79 [1.37–2.36], *p* < 0.001) and solid malignant tumors (OR 1.57 [1.25–1.97], *p* = 0.0001) were also associated with increased risk of polymicrobial bloodstream infection. With regard to the source of infection, abdominal infection was significantly associated with polymicrobial bloodstream infection (OR 1.82 [1.38–2.40], *p* < 0.001), and urinary infection was associated with decreased risk of polymicrobial bloodstream infection (OR 0.53 [0.37–0.74], *p* = 0.0003).

## Discussion

In this nationwide sepsis cohort study, 32.0% of the patients presented with bloodstream infections. Among the patients with bloodstream infections, 11.2% of them had polymicrobial bloodstream infections. This rate of polymicrobial bloodstream infections was similar to reports of previous studies [6, 14]. In this study, the crude mortality of patients with polymicrobial bloodstream infection was higher than those with monomicrobial bloodstream infection. However, according to Cox proportional hazard regression analysis, whether polymicrobial or monomicrobial bloodstream infection was not associated with hospital mortality. In a recent study by Royo-Cebrecos et al., patients with cancer with polymicrobial bloodstream infection had higher early and overall case-fatality rates than those with monomicrobial bloodstream infection [9]. However, in their study, multivariate survival analysis for confounder adjustment was not conducted, so that the result may not suggest actual association of polymicrobial bloodstream infection and hospital mortality. The factors associated with worse outcome in patients with bloodstream infection in our cohort were underlying diseases such as hematologic malignancy, solid malignant tumors, clinical frailty, and pulmonary infection, or primary bacteremia. The inappropriate use of antibiotics and not undergoing source control were also the factors associated with hospital mortality. Consequently, polymicrobial bloodstream infection itself was not the direct cause of hospital mortality. Proper sepsis management such as using appropriate antibiotics and undergoing infection source control was an important factor that improves outcomes of sepsis with bloodstream infection.

In the international guideline for management of sepsis and septic shock, early and appropriate administration of antibiotics is the most effective treatment to reduce mortality in sepsis patients [15]. When selecting the empirical antibiotics in patients with sepsis or septic shock, MRSA coverage is suggested for the patients at high risk of MRSA, and dual gram-negative coverage is suggested for the patients at high risk of MDR microorganism. Additionally, using an antifungal agent is suggested for the patient at high risk of fungal infection. The high risks of MRSA, MDR, or fungal infections are comparatively well understood, but the high risks of polymicrobial bloodstream infections or the antimicrobial strategy for polymicrobial infection in advance is not established yet. In our sepsis cohort, polymicrobial infections were identified in 3.6% of cases, with 20% of these receiving inappropriate antibiotic treatment. Nearly half of the polymicrobial infections involved a combination of gram-positive and gram-negative bacteremia, with an appropriateness rate of 75.2% for antibiotic treatment. Therefore, for patients at high risk of polymicrobial bloodstream infections, such as those with abdominal infections or those in surgical ICUs, the use of broad-spectrum antibiotics effective against both gram-positive and gram-negative bacteria is recommended. Further research is necessary to enhance our understanding and management of polymicrobial infections and to develop more effective antimicrobial strategies.

Intra-abdominal infection was significantly associated with polymicrobial bloodstream infections in this study, whereas urinary infection was associated with a low incidence of polymicrobial bloodstream infections. Several previous studies demonstrated that intra-abdominal infection was the major factor of polymicrobial bloodstream infection [7, 16, 17]. Secondary peritonitis is a major disease of intra-abdominal infection, but infections such as cholecystitis, cholangitis, diverticulitis, and pancreatitis are also broadly included in terms of intra-abdominal infection. The microbiology of intra-abdominal infection is polymicrobial infection in nature because perforation of viscera leads to gastrointestinal flora invasion into the sterile body site and develop intra-abdominal abscess [18, 19]. Therefore, an intra-abdominal abscess is made up of the gastrointestinal flora at the level of the perforation. Especially colon has abundant flora with up to 10^12^ organisms/g of feces. The predominant facultative flora in the colon includes *Escherichia coli*, *Klebsiella pneumoniae*, *Proteus* species, and *Enterococcus* species. In this cohort, the majority of the patients with polymicrobial bloodstream infection had an intra-abdominal infection, and the percentage of cultured bacteria mentioned above were significantly high. For the same reason, polymicrobial infections may be identified more frequently in patients who were admitted to the surgical ICU. In the literature, polymicrobial infection was not associated with mortality in patients with intra-abdominal infection [20]. In the conclusion, this study confirmed that intra-abdominal infection itself is not a factor directly associated with mortality, but rather a predisposing factor of polymicrobial bloodstream infection. In addition, in terms of the underlying diseases, patients with solid malignant tumor patients were more likely to develop polymicrobial bloodstream infections. Several factors were reported to increase the risk of sepsis and bloodstream infection in patients with cancer. The use of immunosuppressant drugs such as chemo agents, long-term central catheters, indwelling urinary catheter, and frequent invasive procedures were the causes of the increasing risk of bloodstream infections [21].

This study is meaningful because it prospectively gathered and analyzed data from patients with sepsis nationwide cohort and it demonstrated the incidence, characteristics, and prognosis of patients with polymicrobial bloodstream infections in comparison to those with monomicrobial bloodstream infection. However, there are several limitations. First, this cohort enrolled patients that were diagnosed with sepsis or septic shock, so the study results did not represent the patients with the entire polymicrobial bloodstream infections. Due to the inclusion of patients with organ dysfunction brought on by infection, it is possible that their mortality and severity will be higher than those patients with the polymicrobial bloodstream infection patients without sepsis or septic shock. Second, due to the nature of data collection of the registry, we did not know the detailed diagnosis, such as cholangitis or liver abscess among intra-abdominal infections. Third, our study period from September 2019 to December 2021 coincided with the COVID-19 pandemic, yet our dataset included only three COVID-19 cases. Given this small number, we believe the pandemic's impact on our findings is minimal. Lastly, while endocarditis is a known significant sepsis source, it was not examined in our research. This exclusion of certain infection sites is a limitation that future studies should address to provide a more comprehensive view of sepsis sources.

## Conclusions

In conclusion, polymicrobial bloodstream infections per se did not increase hospital mortality as compared to monomicrobial infection. Regardless of the number of microbial pathogens present, the most important factors for survival in patients with sepsis with bloodstream infections were the appropriate use of antibiotics and source control.

### Supplementary Information

**Supplementary Material 1. **

## Data Availability

The datasets used and/or analysed during the current study are available from the corresponding author on reasonable request.
