# Supplementary material for: Polymicrobial bloodstream infections per se do not increase mortality compared to monomicrobial bloodstream infections in sepsis patients: a Korean nationwide sepsis cohort study
Source: BMC Infect Dis. 2024 Mar 5;24:285. doi: 10.1186/s12879-024-09130-5 (PMC10913581; doi:10.1186/s12879-024-09130-5)
Supplement: Supplementary file 1 — Supplementary Material 1. [file 12879_2024_9130_MOESM1_ESM.docx]

Polymicrobial bloodstream infections per se do not increase mortality compared to monomicrobial bloodstream infections in sepsis patients: A Korean nationwide sepsis cohort study.

**Supplemental Digital Content**

Appendix 1. Inclusion criteria and enrolment for Korean Sepsis Alliance Registry

Appendix 2. Data collections for sepsis patients

eFigure 1. Types of pathogens and their combinations in polymicrobial bloodstream infections

eTable 1. List and counts of gram-positive bacteria classified as “Others”

eTable 2. List and counts of gram-negative bacteria classified as “Others”

eTable 3. Factors associated with mortality in patients with polymicrobial bloodstream infections by Cox proportional hazards analysis

eTable 4. Factors associated with occurrence of polymicrobial bloodstream infections by logistic regression analysis

Appendix 1. Inclusion criteria and enrolment for Korean Sepsis Alliance Registry

1) Inclusion criteria

- Patient with sepsis aged 19 years or older located in emergency department (ED) or admitted in general ward

2) Exclusion criteria

- Patient aged lower than 19 years

- Sepsis not diagnosed

3) Definition of sepsis

3-1) Community-onset sepsis

- Patient located in the ED and diagnosed with sepsis or septic shock by Sepsis-3 definition
- Time zero: the time patient arrived at ED

3-2) Hospital-onset sepsis

- Patient admitted in a general ward and diagnosed with sepsis or septic shock by Sepsis-3 definition
- Time zero: the time rapid response system (RRS) contact sepsis patient

4) Screening for sepsis

4-1) Community-onset sepsis: fulfilled by the following two criteria:

1. Adult patients aged 19 years or older who underwent blood culture tests in ED
2. Patients satisfy at least two of the following qSOFA criteria

A. Respiratory rate ≥ 22/min

B. Altered mentation

C. Systolic blood pressure (systolic BP) ≦ 100 mmHg

- If it is difficult to evaluate the mental status, patients satisfy at least one of

the respiratory rate or systolic BP criteria

4-2) Hospital-onset sepsis

- Adult patients aged 19 years or older recognized by RRS in participating hospital

5) Diagnosis of sepsis and septic shock according to Sepsis-3 definition:

- Sepsis was diagnosed in patients with organ dysfunction, evidenced by an acute increase in the total SOFA score of 2 points or more, attributable to infection.
- The SOFA score was derived from several parameters, including the PaO2/FiO2 ratio, platelet count, bilirubin level, mean arterial pressure or vasopressor requirement, Glasgow Coma Scale score, and either creatinine level or urine output.
- Septic shock is defined as a state of sepsis characterized by persistent hypotension requiring vasopressors to maintain a mean arterial pressure (MAP) of 65 mmHg or higher, and a serum lactate level greater than 2 mmol/L, despite adequate volume resuscitation.

6) Enrollment of patients

- Among the screened patients, patients who have met the definition of Sepsis-3 definition were registered in Korean Sepsis Alliance Registry

Appendix 2. Data collections for sepsis patients

The following information from electronic medical records were collected:

Demographic data including age, sex, height, weight, body mass index (BMI), underlying comorbidities, clinical frailty scale, Eastern Cooperative Oncology Group (ECOG) performance score, disease severity scores of Sequential Organ Failure Assessment (SOFA) score, Simplified Acute Physiology Score (SAPS) III, hospital outcomes including death, hospital length of stay, ICU admission and ICU length of stay, received treatment during hospitals including antibiotics, vasopressor, fluid resuscitation, mechanical ventilation or hemodialysis, serial physiologic data (including blood pressure, pulse, and respiratory rates), temperature, percutaneous oxygen saturation, and laboratory findings including arterial blood gas analysis, complete blood count, and chemistry

eFigure 1. Types of pathogens and their combinations in polymicrobial bloodstream infections


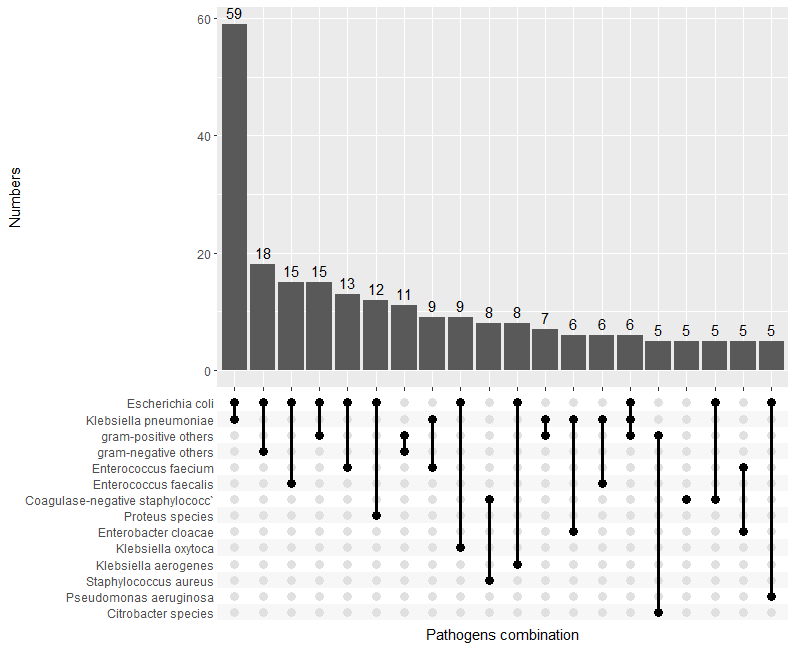


eTable 1. List and counts of Gram-positive bacteria classified as “Others”

| Names | Monomicrobial BSI, counts (n) | Polymicrobial BSI, counts (n) |
| --- | --- | --- |
| Actinomyces odontolyticus | 1 | 0 |
| Alloiococcus species | 2 | 0 |
| Anaerococcus lactolyticus | 1 | 0 |
| Arthrobacter species | 1 | 0 |
| Atopobium vaginae | 1 | 0 |
| Bacillus cereus | 3 | 0 |
| Bacillus spp. | 8 | 0 |
| Bifidobacterium spp. | 1 | 0 |
| Brevibacterium casei | 1 | 0 |
| Clostridium Perfringens | 14 | 4 |
| Clostridium spp. | 10 | 8 |
| Corynebacterium species | 8 | 0 |
| Cutibacterium acnes | 1 | 0 |
| Eggerthella lenta | 1 | 0 |
| Enterococcus spp. | 14 | 24 |
| Kocuria species | 1 | 0 |
| Lactobacillus species | 2 | 0 |
| Listeria monocytogenes | 1 | 0 |
| Microbacterium spp. | 3 | 0 |
| Micrococcus luteus | 2 | 0 |
| Parvimonas micra | 2 | 0 |
| Peptoniphilus spp | 3 | 0 |
| Peptostreptococcus micros | 1 | 0 |
| Propionibacterium spp. | 1 | 0 |
| Robinsoniella peoriensis | 1 | 0 |
| Streptococcus dysgalactiae | 14 | 1 |
| Streptococcus species, viridans group | 66 | 41 |
| Turicella otitidis | 1 | 0 |
| Weissella confusa | 1 | 0 |
| Gemella haemolysans | 0 | 1 |
| Granulicatella adiacens | 0 | 1 |
| Parvimonas micra | 0 | 1 |
| Solobacterium moorei | 0 | 1 |
| Not descripted | 20 | 0 |
| Total | 186 | 82 |

eTable 2. List and counts of Gram-negative bacteria classified as “Others”

| Names | Monomicrobial BSI, counts (n) | Polymicrobial BSI, counts (n) |
| --- | --- | --- |
| Achromobacter xylosoxidans | 5 | 4 |
| Acinetobacter nosocomialis | 3 | 0 |
| Aeromonas spp. | 10 | 16 |
| Bacteroides fragilis | 16 | 5 |
| Bacteroides spp. | 16 | 4 |
| Burkholderia cepacia | 2 | 0 |
| Buttiauxella agrestis | 1 | 0 |
| Campylobacter fetus | 1 | 0 |
| Chryseobacterium spp. | 1 | 2 |
| Eikenella corrodens | 1 | 1 |
| Elizabethkingia spp. | 2 | 1 |
| Enterobacter spp. | 1 | 1 |
| Fusobacterium spp. | 5 | 1 |
| Klebsiella variicola | 1 | 1 |
| Kluyvera ascorbata | 1 | 0 |
| Moraxella spp. | 1 | 1 |
| Morganella morganii | 10 | 9 |
| Myroides spp. | 1 | 0 |
| Neisseria elongata | 1 | 0 |
| Neisseria animaloris | 1 | 0 |
| Ochrobactrum spp. | 2 | 0 |
| Odoribacter splanchnicus | 2 | 0 |
| Pantoea spp. | 2 | 0 |
| Plesiomonas shigelloides | 1 | 0 |
| Prevotella intermedia | 1 | 0 |
| Providencia spp. | 10 | 2 |
| Raoultella spp. | 7 | 3 |
| Roseomonas mucosa | 1 | 0 |
| Salmonella spp. | 13 | 0 |
| Serratia spp. | 3 | 0 |
| Shewanella putrefaciens | 1 | 0 |
| Sphingomonas spp. | 2 | 0 |
| Vibrio vulnificus | 3 | 0 |
| Veillonella parvula | 0 | 1 |
| Helicobacter pylori | 0 | 1 |
| Not descripted | 9 | 7 |
| Total | 137 | 60 |

eTable 3. Factors associated with mortality in patients with polymicrobial bloodstream infections by Cox proportional hazards analysis

| Variable | Univariate | | | Multivariate | | |
| --- | --- | --- | --- | --- | --- | --- |
|  | HR | 95%CI | P-value | HR | 95%CI | P-value |
| Age | 1.01 | 1.00, 1.03 | 0.051 | 1.01 | 1.00, 1.03 | 0.062 |
| Sex, male | 0.99 | 0.71, 1.37 | >0.9 |  |  |  |
| BMI | 0.96 | 0.92, 1.00 | 0.079 |  |  |  |
| Community-onset sepsis | 1.21 | 0.84, 1.75 | 0.3 |  |  |  |
| Medical department | 2.42 | 1.11, 5.30 | 0.027 |  |  |  |
| Surgical department | 0.41 | 0.19, 0.90 | 0.027 |  |  |  |
| Cardiovascular disease | 1.00 | 0.68, 1.48 | >0.9 |  |  |  |
| Chronic lung disease | 1.14 | 0.65, 2.02 | 0.6 |  |  |  |
| Chronic neurological disease | 1.01 | 0.69, 1.47 | >0.9 |  |  |  |
| Chronic liver disease | 1.10 | 0.69, 1.74 | 0.7 |  |  |  |
| Diabetes | 1.05 | 0.76, 1.45 | 0.8 |  |  |  |
| Chronic kidney disease | 1.15 | 0.73, 1.80 | 0.6 |  |  |  |
| Connective tissue disease | 1.74 | 0.85, 3.56 | 0.13 |  |  |  |
| Immunocompromised | 1.00 | 0.53, 1.88 | >0.9 |  |  |  |
| Hematological malignancies | 1.55 | 0.93, 2.57 | 0.092 |  |  |  |
| Solid malignant tumors | 1.16 | 0.84, 1.59 | 0.4 |  |  |  |
| Charlson comorbidity index | 1.08 | 1.01, 1.15 | 0.017 |  |  |  |
| ECOG score >=2 | 2.67 | 1.81, 3.95 | <0.001 | 1.65 | 0.98, 2.76 | 0.059 |
| CFS score | 1.22 | 1.12, 1.31 | <0.001 |  |  |  |
| Non-fragile (1-4) | 1 |  |  | 1 |  |  |
| Mild-to-mod fragile (5–6) | 1.64 | 1.02, 2.65 | 0.042 | 1.27 | 0.73, 2.21 | 0.391 |
| Severe fragile (7–9) | 2.73 | 1.86, 3.99 | <0.001 | 1.73 | 1.03, 2.90 | 0.040 |
| Source of infection |  |  |  |  |  |  |
| Pulmonary | 1.86 | 1.30, 2.67 | <0.001 |  |  |  |
| Abdominal | 0.63 | 0.45, 0.86 | 0.004 |  |  |  |
| Urinary | 0.48 | 0.27, 0.85 | 0.012 | 0.38 | 0.21, 0.68 | 0.001 |
| Skin and soft tissue | 1.61 | 0.86, 3.02 | 0.13 |  |  |  |
| Catheter-related | 1.18 | 0.48, 2.89 | 0.7 |  |  |  |
| Systemic infections | 2.52 | 1.61, 3.93 | <0.001 | 2.01 | 1.26, 3.20 | 0.003 |
| Neurologic | 0.57 | 0.08, 4.07 | 0.6 |  |  |  |
| Initial septic shock | 1.14 | 0.80, 1.62 | 0.5 |  |  |  |
| Inappropriate use of antibiotics | 1.46 | 1.01, 2.11 | 0.041 |  |  |  |
| Source control | 0.27 | 0.16, 0.46 | <0.001 | 0.32 | 0.19, 0.54 | <0.001 |
| MDR pathogen | 1.11 | 0.99, 1.25 | 0.087 |  |  |  |

HR, hazard ratio; CI, confidence interval; BMI, body mass index; ECOG, European Cooperative Oncology Group; CFS, Clinical Frailty scale; MDR, multidrug resistant

eTable 4. Factors associated with occurrence of polymicrobial bloodstream infections by logistic regression analysis

| Variable | Univariate | | | Multivariate | | |
| --- | --- | --- | --- | --- | --- | --- |
|  | OR | 95% CI | P-value | OR | 95% CI | P-value |
| Age | 1.00 | 0.99, 1.01 | 0.7 |  |  |  |
| Sex, male | 1.20 | 0.98, 1.47 | 0.080 |  |  |  |
| BMI | 0.97 | 0.95, 1.00 | 0.034 | 0.98 | 0.95, 1.00 | 0.0992 |
| Community onset sepsis | 0.81 | 0.64, 1.03 | 0.086 |  |  |  |
| Medical department | 0.52 | 0.34, 0.82 | 0.004 | 0.96 | 0.72, 1.29 | 0.7971 |
| Surgical department | 1.92 | 1.21, 2.98 | 0.004 | 1.76 | 1.17, 2.64 | 0.0063 |
| Comorbidity, n(%) |  |  |  |  |  |  |
| Cardiovascular disease | 0.93 | 0.72, 1.18 | 0.6 |  |  |  |
| Chronic lung disease | 0.86 | 0.59, 1.20 | 0.4 |  |  |  |
| Chronic neurological disease | 1.24 | 0.98, 1.56 | 0.072 | 1.79 | 1.37, 2.36 | <0.001 |
| Chronic liver disease | 0.95 | 0.68, 1.29 | 0.8 |  |  |  |
| Diabetes | 1.11 | 0.90, 1.37 | 0.3 |  |  |  |
| Chronic kidney disease | 1.02 | 0.75, 1.37 | 0.9 |  |  |  |
| Connective tissue disease | 1.35 | 0.73, 2.32 | 0.3 |  |  |  |
| Immunocompromised | 1.56 | 0.95, 2.43 | 0.064 |  |  |  |
| Hematological malignancies | 0.86 | 0.57, 1.25 | 0.4 |  |  |  |
| Solid malignant tumors | 1.92 | 1.57, 2.36 | <0.001 | 1.57 | 1.25, 1.97 | 0.0001 |
| Charlson comorbidity index | 1.07 | 1.04, 1.11 | <0.001 |  |  |  |
| ECOG>=2 | 1.19 | 0.97, 1.47 | 0.11 |  |  |  |
| CFS score | 0.12 | 0.10,0.14 | <0.001 |  |  |  |
| Non-frail (1-4) | 1 |  |  | 1 |  |  |
| Mild-to-moderately frail (5–6) | 1.07 | 0.81, 1.41 | 0.6 | 1.26 | 0.94, 1.68 | 0.1257 |
| Severely frail (7-9) | 1.22 | 0.97, 1.53 | 0.084 | 1.37 | 1.06, 1.78 | 0.0155 |
| Source of infection |  |  |  |  |  |  |
| Pulmonary | 0.68 | 0.52, 0.86 | 0.002 | 0.76 | 0.55, 1.05 | 0.0907 |
| Abdominal | 2.31 | 1.89, 2.84 | <0.001 | 1.82 | 1.38, 2.40 | <0.001 |
| Urinary | 0.45 | 0.34, 0.58 | <0.001 | 0.53 | 0.37, 0.74 | 0.0003 |
| Skin and soft tissue | 1.04 | 0.62, 1.65 | 0.9 |  |  |  |
| Catheter-related | 0.92 | 0.44, 1.70 | 0.8 |  |  |  |
| Systemic infections | 0.80 | 0.55, 1.12 | 0.2 |  |  |  |
| Neurologic | 2.12 | 0.60, 5.87 | 0.2 |  |  |  |

OR, odds ratio; CI, confidence interval; BMI, body mass index; ECOG, European Cooperative Oncology Group; CFS, Clinical Frailty scale
